# A Sequential Micro-Immunotherapy Medicine Increases Collagen Deposition in Human Gingival Fibroblasts and in an Engineered 3D Gingival Model under Inflammatory Conditions

**DOI:** 10.3390/ijms241310484

**Published:** 2023-06-22

**Authors:** Maria del Mar Ferrà-Cañellas, Marta Munar-Bestard, Ilaria Floris, Joana Maria Ramis, Marta Monjo, Laura Garcia-Sureda

**Affiliations:** 1 Group of Cell Therapy and Tissue Engineering, Research Institute on Health Sciences (IUNICS), University of the Balearic Islands, 07122 Palma de Mallorca, Spain; mar.ferra@labolife.com (M.d.M.F.-C.); marta.munar@uib.es (M.M.-B.); marta.monjo@uib.es (M.M.); 2 Preclinical Research Department, Labo’Life España, 07330 Consell, Spain; 3 Balearic Islands Health Research Institute (IdISBa), 07122 Palma de Mallorca, Spain; 4 Preclinical Research Department, Labo’Life France, 44000 Nantes, France; ilaria.floris@labolife.com

**Keywords:** micro-immunotherapy, periodontitis, human gingival fibroblast, inflammation, in vitro, low doses therapy, immunomodulation, BMP4, sequential medicine, prostaglandin E2, 3D gingival model

## Abstract

Periodontal therapies use immune mediators, but their side effects can increase with dosage. Micro-immunotherapy (MI) is a promising alternative that employs immune regulators at low and ultralow doses to minimize adverse effects. In this study, the effects of 5 capsules and the entire 10-capsule sequence of the sequential MI medicine (MIM-seq) were tested in two in vitro models of periodontitis. Firstly, human gingival fibroblasts (hGFs) exposed to interleukin (IL)-1β to induce inflammation were treated with five different capsules of MIM-seq for 3 days or with MIM-seq for 24 days. Subsequently, MIM-seq was analyzed in a 3D model of human tissue equivalent of gingiva (GTE) under the same inflammatory stimulus. Simultaneously, a non-IL-1β-treated control and a vehicle were included. The effects of the treatments on cytotoxicity, collagen deposition, and the secreted levels of IL-1α, IL-6, prostaglandin E2 (PGE2), matrix metalloproteinase-1 (MMP-1), and tissue inhibitor of metalloproteinases-1 (TIMP-1) were evaluated. None of the tested items were cytotoxic. The complete sequence of MIM-seq decreased PGE2 release and restored collagen deposition levels induced by IL-1β treatment in hGFs exposed to IL-1β. MIM-seq treatment restored collagen production levels in both models. These promising preclinical findings suggest that MIM-seq should be further investigated for periodontitis treatment.

## 1. Introduction

Periodontal diseases constitute the clinical expression of a group of pathologies that affect a large percentage of the world population, which have in common the inflammatory destruction of the supporting tissues of the teeth. The inflammatory process may affect the gingival connective tissue, the periodontal ligament, or the alveolar bone and, in severe cases, such as those of periodontitis, can result in the loss of periodontal attachment and tooth loss [1,2].

The initiation and progression of periodontitis are mediated by multiple etiological factors; however, one of the most relevant is related to local inflammation and an increase in several proinflammatory mediators [3,4]. Thus, at the site of periodontitis, there is an imbalance of the cytokine and chemokine network characterized by increased levels of proinflammatory cytokines such as interleukin (IL)-1β, IL-6, and IL-12 and tumor necrosis factor (TNF)-α, and decreased levels of regulatory/anti-inflammatory cytokines such as IL-4, IL-1 receptor antagonist (RA), IL-10, and induced protein-10 [5,6].

Anabolic bone growth factors such as platelet-derived growth factor (PDGF), members of the transforming growth factor-beta (TGF-β) family such as Bone Morphogenetic Proteins (BMPs) (BMP-2, BMP-4, and BMP-7), and insulin growth factor (IGF) are also involved in the homeostasis of the immune response and normal metabolism of the periodontal tissues. In addition to the typically proinflammatory cytokines mentioned above, patients suffering from periodontitis also show imbalances in these growth factors [7,8,9,10].

The main goal of periodontal therapy is to reduce the inflammatory response and enhance periodontal tissue regeneration by stimulating the regeneration of damaged tissues [1,2]. Therefore, therapies such as guided bone regeneration or tissue-stimulating protein techniques, which involve applying endogenous growth factors derived from plasma and platelets or other immunomodulatory mediators, are increasingly being studied [11,12,13,14].

In spite of the huge potential of immunomodulatory treatments, caution must be exerted when using these mediators, as research shows that they have different side effects at the clinical level depending on their dose [15,16,17]. A clear example of this is the case of BMP-2, as the incidence of associated adverse effects has been observed to increase concomitantly with increasing the dose used [8]. Therefore, lowering their dose could be a good strategy to minimize the side effects. The therapeutic approach of micro-immunotherapy (MI), which uses low doses (LDs) and ultralow doses (ULDs) of bioactive molecules and immune factors, could be of great interest in this context. Previous preclinical studies have already shown the potential of MI in the context of inflammatory diseases, such as arthritis or allergy [18,19,20,21,22], and their anti-inflammatory effects have been observed both at local and systemic levels in vivo [18,20]. Moreover, the recently published in vitro study on the unitary MI medicine BMP-4 (5 CH) shows encouraging results, specifically in the context of periodontal disease [23].

The investigational product of this preclinical study is a sequential MIM (hereafter referred to as MIM-seq in this manuscript) composed of 10 different capsules, each of them with a specific combination of immune mediators, such as BMP-4, in LD and ULD, impregnated on sucrose–lactose pillules (also called globules) for oromucosal administration [22]. The composition of the sequential MIM is expressed in centesimal Hahnemannian (CH) dilutions. The active substances employed in MIM-seq could be classified by their potential targets and hypothetical mode of action: (a) Substances which can help decrease and resolve inflammation, such as the cytokines IL-1 and IL-6; TNFα in ULD [18,19,22]; or TGF-β in LD [24]. (b) Substances acting on the extracellular matrix (ECM) and bone remodeling, mainly LD of BMP-2 and BMP-4 [24,25,26] and also ULD of IGF-1 [27,28,29] and Granulocyte-Macrophage Colony-Stimulating Factor (GMCSF) [30].

The complete list of active substances defining the MIM-seq composition is the following: IL-1β (17 CH), IL-6 (17 CH), IL-11 (15 CH), IGF-1 (9 CH), GMCSF (15 CH), TGF-β (5 CH), TNF-α (17 CH), BMP-2 (5 CH), and BMP-4 (5 CH); and nucleic-acid-based-molecules such as DNA (5 CH), RNA (5 CH), and specific nucleic acids (SNA) such as SNA^®^-OSTEOa-02 (18 CH) and SNA^®^-OSTEOb-02 (18 CH), targeting human leukocyte antigen (HLA) I and II, respectively, and the fluoride derivative natrum silico fluoricum (Na2SiF6) (3 CH) (see the first column of Table 1).

Given the complexity of MIM-seq formulation, our approach was to start with an in vitro study to test five different capsules composing MIM-seq independently. The five tested capsules, referred to as MIM-1, MIM-2, MIM-3, MIM-4, and MIM-5 (see their composition in Table 1), were investigated in vitro using a preclinical model of periodontitis. The cellular model used in the present study consists of human gingival fibroblasts (hGFs) exposed to 1 ng/mL of IL-1β as an inflammatory stimulus before the incubation with the tested MIMs. Then, the effects of the entire MIM-seq were assessed in the same model, and a three-dimensional model (3D) of oral mucosa also treated with 1 ng/mL of IL-1β was used to confirm the obtained results.

This study had, therefore, two main objectives. Firstly, we investigated the anti-inflammatory effects of five of the capsules composing the medicine after 3 days of treatment, setting up a short-time experiment to analyze the independent effect of each of the combinations of immunomodulators, analyze their cytotoxicity, and see their short-term effect, as has already been carried out in other studies [18,21,23,31,32]. Then, the entirety of the MIM-seq effects were assessed in two long-time experiments to investigate the cumulative effect of MIM-seq in a two-dimensional model of gingival fibroblasts and a three-dimensional model of oral mucosa. In both cases, the cells received the complete 10-capsule sequence in a manner that reproduced the human-intended posology.

## 2. Results

### 2.1. Short-Time Experiments

**Figure 1:**
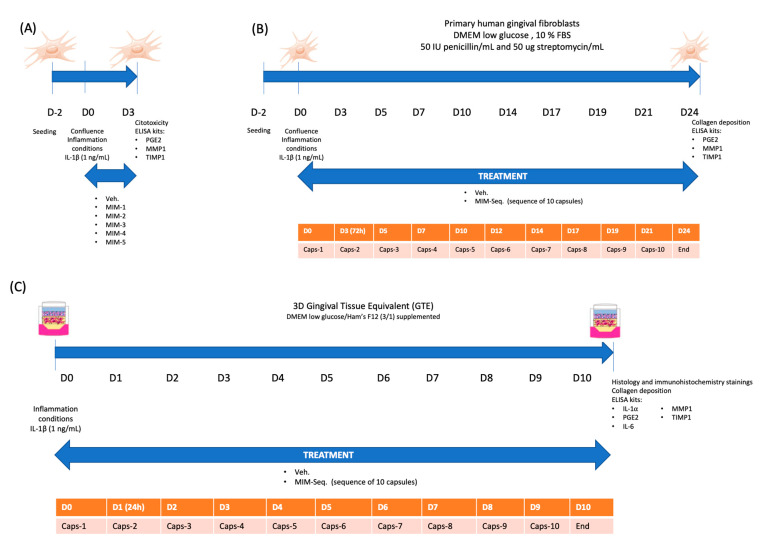
Representative experimental protocol. Primary human fibroblasts, at confluence (D0), exposed to 1 ng/mL of IL-1β as an inflammatory inducer. (**A**) Short-time experiment (3 days), treatment with MIM-1, MIM-2, MIM-3, MIM-4, MIM-5, or vehicle (Veh.) from day 0 (D0) to day 3 (D3). Cytotoxicity, PGE2, MMP-1, and TIMP-1 levels analyzed at D3. (**B**) Long-time experiment (24 days) treatment with MIM-seq (10 capsules), or the Veh. Collagen deposition, PGE2, MMP-1, and TIMP-1 levels analyzed at day 24. (**C**) 3D gingival tissue equivalent (GTE) experiment (10 days) treatment with MIM-seq (sequence of 10 capsules), or the Veh. Histology and immunohistochemistry staining, IL-1α, PGE2, IL-6, MMP-1, and TIMP-1 levels were analyzed at day 10.

**Figure 2:**
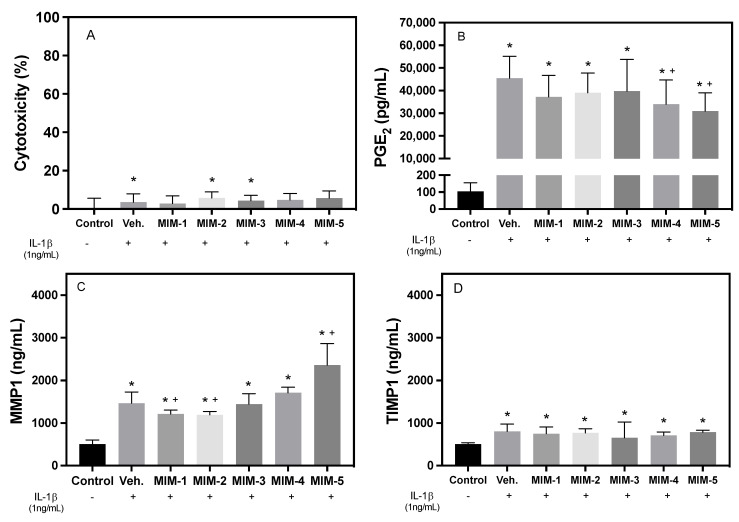
Effects of each of the 5 capsules composing the sequential MIM assessed after 3 days of treatment in hGF cells inflamed with IL-1β. (**A**) Cytotoxicity. Results are shown relative to LDH activity in cell culture media. Negative control = 0% cell death, positive control = 100% cell death (1% nonionic surfactant). (**B**) PGE2, (**C**) MMP-1, and (**D**) TIMP-1 protein released to cell culture media. Data represent media ± SEM of six sample replicates (*n* = 6) for each group. A and B were compared using ANOVA and LSD as post hoc, while C and D were compared using the Kruskal–Wallis test. +: Cells treated with IL-1β, -: Cells not treated with IL-1β. Statistically significant differences were considered for *p* < 0.05 and are represented with * compared to control and + compared to vehicle/inflammatory control.

First of all, five capsules composing MIM-seq (MIM-1, MIM-2, MIM-3, MIM-4, and MIM-5) were analyzed individually after 3 days of treatment for their effects on cell viability (Figure 1A). In the cytotoxicity graph (Figure 2A), only the vehicle, MIM-2, and MIM-3 showed significant changes compared to the uninflamed, untreated control. However, all the tested treatments showed values below 10%, confirming that neither the vehicle nor the treatments were cytotoxic to fibroblasts at the tested concentration.

To evaluate the potential anti-inflammatory effects of the five tested capsules, the secreted levels of PGE2 were measured and compared with the vehicle/inflammatory control. Figure 1B shows the increase in PGE2 in all the treated groups compared to the non-IL-1β-treated control, validating the inflammatory model. The secreted levels of PGE2 were between 30,000 pg/mL and more than 50,000 pg/mL, while for the non-IL-1β-treated cells, they were found to be around 100 pg/mL. When looking at the effect of each capsule compared to the vehicle, it can be seen that MIM-4 and MIM-5 were able to reduce significantly the levels of PGE2: MIM-4 induced a 25.3% (*p*-value: 0.042) reduction and MIM-5 induced a 32.0% reduction compared to the vehicle/inflammatory control (*p*-value: 0.012), respectively. MIM-1, MIM-2, and MIM-3 did not show significant differences from the vehicle treatment.

Figure 2C shows an increased release of MMP-1 into the cell culture medium in all the treated groups compared to the non-IL-1β-treated control. Indeed, all groups reached values 2 to 3 times higher than the control. Concerning the effects of the five tested MIMs, MIM-1 and MIM-2 showed statistically significant lower values compared to the vehicle/inflammatory control. Conversely, MIM-5 was found able to significantly increase MMP-1 release compared to the vehicle/inflammatory control. The assessed levels of TIMP-1 revealed that its secretion was slightly increased by IL-1β in all groups after 3 days compared to the non-IL-1β-treated control (Figure 2D). In this case, according to the results, none of the tested MIM treatments influenced TIMP-1 secretion compared to the vehicle/inflammatory control.

### 2.2. Long-Time Experiments

Subsequently, the effects of the complete series of 10 capsules composing the sequential treatment of the MIM were assessed in long-term experiments: firstly, in the model of gingival fibroblast (Figure 1B), and secondly in a 3D cell culture model of human tissue equivalent of gingiva (GTE) under the same inflammatory stimulus (Figure 1C).

**Figure 3:**
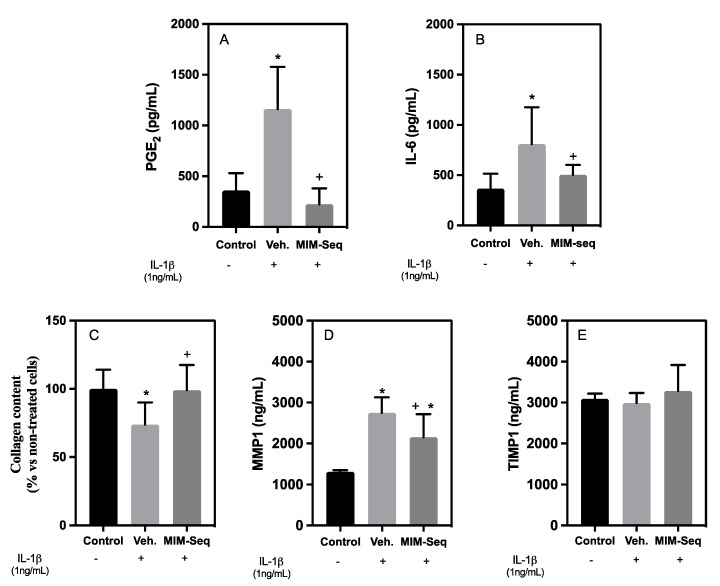
Effects of the sequential medicine MIM-seq after 24 days of treatment in hGF cells inflamed with IL-1β. Inflammation was evaluated with (**A**) PGE2 and (**B**) IL-6 release of GTE media culture. (**C**) Collagen deposition of hGF cells. (**D**) MMP-1 and (**E**) TIMP-1 protein released to cell culture media. Data represent the media ± SEM of six sample replicates (*n* = 6) for each group. Results from A and C were compared using ANOVA and LSD as post hoc, while (**B**,**D**,**E**) were compared using the Kruskal–Wallis test. +: Cells treated with IL-1β, -: Cells not treated with IL-1β. Statistically significant differences were considered for *p* < 0.05 and are represented with * compared to control and + compared to vehicle.

In the case of hGF cells, the PGE2 levels of vehicle/inflamed cells after 24 days of treatment were significantly higher than the non-IL-1β-treated control, as expected. The cumulative effect of the MIM-seq treatment showed a significant reduction in PGE2 levels compared to the vehicle (Figure 3A), with similar levels to those of the non-IL-1β-treated control cells. A second inflammatory marker was evaluated with IL-6 release (Figure 3B). Vehicle/inflamed cells were significantly higher compared to the non-IL1𝛽-treated control after 10 days of treatment. Meanwhile, MIM-seq treatment showed a significant reduction in IL-6 levels compared to the vehicle, with similar levels to the uninflamed control.

After 24 days of treatment, the vehicle-inflamed cells showed a significant reduction in their collagen levels compared to the control. Interestingly, MIM-seq treatment increased collagen content compared to the vehicle, and their levels reached values similar to those of the non-IL-1β-treated control (Figure 3C).

MMP-1 secretion was found to be significantly decreased in the MIM-seq-treated cells compared to the vehicle, but those levels did not reach the values observed in the control (Figure 3D). Regarding the results of TIMP-1 levels, as shown in Figure 3E, neither the vehicle nor the MIM-seq influenced TIMP-1 secretion compared to the non-IL-1β-treated control group.

**Figure 4:**
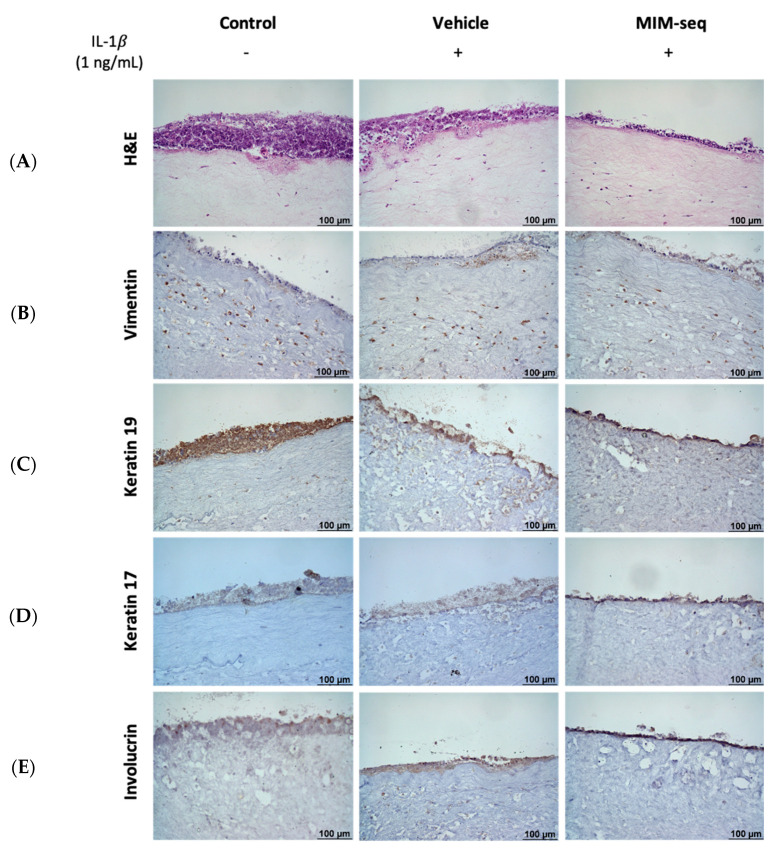
Histologic and immunohistochemistry characterization after 10 days of treatment of GTE culture model inflamed with IL-1β. (**A**) GTE stained with H&E (hematoxylin and eosin). (**B**) Vimentin expression (a marker for fibroblasts). (**C**) Keratin 19 expression (a marker for epithelial differentiation). (**D**) Keratin 17 expression (a marker for epithelial differentiation). (**E**) Involucrin expression (a marker for epithelial differentiation). +: Cells treated with IL-1β, -: Cells not treated with IL-1β. Pictures were taken at a magnification of 200×. Scale bar is represented on images.

For the 3D model, histological characterization and validation of a correct differentiation of GTEs were observed after the complete 10-capsule sequence treatment for all the experimental groups (Figure 4). Hematoxylin and eosin staining revealed a multilayered epithelium containing a collagen matrix embedded with fibroblasts, similar to the native oral mucosa (Figure 4A). Immunohistochemistry characterization showed expression of vimentin, a differentiation marker for fibroblasts (Figure 4B), and expression of different markers for the epithelial structure of keratinocytes, such as Keratin 19 (Figure 4C), Keratin 17 (Figure 4D), and Involucrin (Figure 4E), which confirmed the differentiation of the multilayer epithelial structure of keratinocytes, confirming the resemblance to native oral mucosa, with no observable differences between the experimental groups.

**Figure 5:**
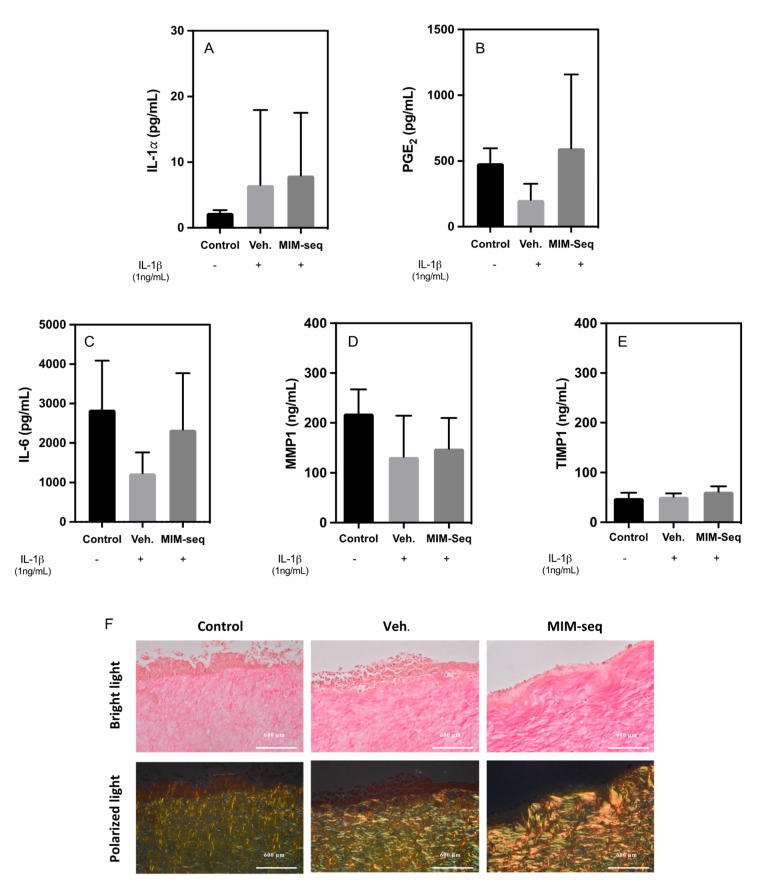
Effects of the sequential medicine MIM-seq after 10 days of treatment in GTE 3D culture model inflamed with IL-1β. (**A**) Tissue viability was measured with the release of IL-1𝛼 of GTE culture. Inflammation was evaluated with (**B**) PGE2 and (**C**) IL-6 release of GTE media culture. (**D**) MMP1 and (**E**) TIMP1 protein release of GTE culture. (**F**) Representative images of Sirius Red F3BA for collagen staining. Larger collagen fibers detected as bright yellow or orange under polarized light microscopy, while the thinner ones including reticular fibers are detected as green. Scale bar is represented on images. Data represent the media ± SEM of six sample replicates (*n* = 6) for each group. +: Cells treated with IL-1β, -: Cells not treated with IL-1β. Results from A were compared using Kruskal–Wallis test, while (**B**–**E**) were compared using ANOVA and LSD as post hoc. No statistically significant differences were found between groups.

Cytotoxicity for 3D GTEs was evaluated through the liberation of IL-1𝛼 (Figure 5A). No statistical differences were found between the groups, although there were higher values for the vehicle and MIM-seq compared to the non-IL-1β-treated control tissues.

PGE2 (Figure 5B) and IL-6 (Figure 5C) liberation were evaluated to assess the inflammatory response of 3D model GTEs to MIM-seq. The results showed no significant differences between the groups, with high variability in the results.

Finally, in relation to ECM organization, there were no significant differences in the release levels of MMP1 (Figure 5D) and TIMP1 (Figure 5E). However, larger fibers of collagen, shown as bright yellow/orange fibers through Sirius Red staining observed under polarized light microscopy, were observed for the MIM-seq treatment group.

## 3. Discussion

In this study, the effect of several combinations of active substances in LD and ULD was analyzed in an in vitro model of periodontitis, the same as that previously used when we assessed the effect of the unitary MIM BMP-4 [23]. As such, five different capsules (MIM-1, MIM-2, MIM-3, MIM-4, and MIM-5) were tested in a short-time experiment, while the effect of entire sequence of MIM-seq was assessed in long-time experiments.

The cytotoxicity associated with immune-mediated treatments can be defined in different ways. In this study, cytotoxicity was measured by determining the increase in LDH activity in the culture medium after a 3-day treatment of the hGFs. The cytotoxicity results showed good biocompatibility of both the vehicle and the MIM treatments in this cell model, as also obtained in other cytotoxicity studies with MIMs such as BMP4 (5CH) [23] or others [18,21,31].

Evaluation of the cytotoxicity in a long-term MIM-seq treatment was evaluated in the GTE tissues through the liberation of IL-1𝛼 to culture medium, known as a viability marker in long-term treatments, assessing cellular health and detecting potential adverse effects [33]. IL-1α levels can indicate cellular stress, inflammation, and disrupted signaling pathways [34,35]. Therefore, although the results manifested high variability, long-time treatment with MIM-seq or with the vehicle showed also good biocompatibility in a more complex system. In addition, the H&E histological sections of the tissues and immunostaining results confirmed the viability of the tissues, with no tissue disruption after long-time treatments, presenting a good multilayer epithelial structure and expression of typical markers of the oral mucosa, as previously discussed in this oral mucosa model [23].

PGE2 is a potent lipid mediator produced by the metabolism of arachidonic acid via the cyclooxygenase (COX) pathway [36], and its secretion can be induced by proinflammatory stimuli such as IL-1β [37,38,39]. Therefore, after 3 days, all cells that were treated with IL-1β showed an increase in PGE2 secretion compared to the non-IL-1β-treated control cells, as reported in other studies that used this same model [2,23]. Amongst the different MIM compositions tested, MIM-5 was the treatment that showed the greatest capacity to dampen the effect of IL-1β, as we observed the lowest PGE2 values. MIM-5 differs from the rest of the treatments mainly by the presence of a LD of TGF-β. TGF-β, like BMP-2 and BMP-4, is a growth factor and a pleiotropic cytokine with potent regulatory roles in the context of inflammation [24]. TGF-β (5CH) could have exerted an anti-inflammatory action that, at least partially, could explain the results. As MIM-5 also includes five more biomolecules in its composition, we cannot exclude the effect being mediated by the others, especially IL-6 and IL-11 at an ULD. MIM-4 also induced a significant reduction in PGE2 values compared to the vehicle/inflamed control. Looking at MIM-4’s composition, it can be hypothesized that its anti-inflammatory effect could be mediated by the presence of IL-1 and TNF-α at ULDs, whose anti-inflammatory effects have been shown in other studies [18,19,22,40,41].

Matrix metalloproteinase (MMP) enzymes play a central role in the maintenance and remodeling of the extracellular matrix. MMP-1 and MMP-3 have been demonstrated to play an important role in periodontitis. MMP-1 can initiate extracellular matrix destruction and cooperates with other MMPs in collagen degradation [42]. The activity of MMPs is controlled by proteolytic activation, production regulation, and neutralization by TIMP-1. An imbalance of MMPs over their inhibitors results in a fibrolytic situation [43]. Moreover, previous studies have shown that IL-1 upregulates the levels of MMP-1 when added to cells in culture [42,44].

Focusing on the effect on ECM turnover at 3 days, there were changes in MMP-1 production that can be attributed to the presence of IL-1β. Concerning the effect of the tested MIM formulations, MIM-1 and MIM-2 significantly, but slightly, reduced MMP-1 levels, while MIM-5 significantly increased MMP-1 levels compared to the vehicle, suggesting that those formulations might influence the degradation of the ECM [43]. Further studies on other MMPs are needed to complete those results and understand the cellular effects of those MIMs on ECM turnover. According to the results obtained with TIMP-1, the inflammatory stimulus used in the in vitro model slightly increased the levels of TIMP-1, while none of the tested capsules displayed an effect.

In brief, after 3 days, the different combination of BMP4 (5CH) with other immunomodulators resulted in the different MIM-seq capsules acting differently on hGFs, both in terms of inflammatory parameters and parameters related to changes in the ECM.

Subsequently, the cumulative effect of a complex and sequential MIM formula was investigated. For this purpose, the treatment MIM-seq, consisting of a sequence of 10 capsules, was tested in long-term experiments, lasting 24 days for hGF cell culture and 10 days for GTE oral tissues.

For hGF, the anti-inflammatory effect of MIM-seq was shown in the PGE2 and IL-6 release results, where it can be seen that the MIM-seq treatment was capable of reversing the inflammation caused by the application of IL-1β, recovering the uninflamed control levels. Thus, the results show a cumulative effect due to the action of the entire sequence and the complete formulation employed. The results are sustained by previous evidence as ULDs of IL-1 and TNF-α have been shown to display anti-inflammatory effects in vitro [21]. In addition, it is important to consider the previous work on the action of BMP-4 (5 CH), which is one of the active substances listed in MIM-seq composition, and it was able to increase collagen deposition and reduce PGE2 release in the same in vitro model of periodontitis [23].

However, the potential anti-inflammatory effect of MIM-seq could not be confirmed in the 3D system of oral mucosa. Differences in the complexity of cell cultures could explain variations in the response to the long-time treatment of MIM-seq. GTEs are composed of gingival fibroblasts embedded into a complex matrix of collagen, and a well-organized keratinocyte layer, with the interaction of two cell lines that participate in the inflammatory response. In addition, the monolayer of gingival fibroblasts of the 2D experiment is primary cells, while the gingival fibroblasts of GTEs are an immortalized cell line. Based on the differences in hGF monolayers and 3D gingiva culture models, it is evident that the use of 3D models could be necessary for studying the effects of specific molecules. Although the interaction of immune system cells is still missing, their use provides a higher degree of complexity closer to the in vivo situation [45,46,47].

Another of the parameters analyzed at 24 days was the collagen content of the hGFs, which is important for periodontitis as its destruction causes the gingiva to recede, with less attachment to the tooth, and gives rise to the typical periodontal pocket, where bacteria that damage accumulate, damaging the gingiva and leading to tooth loss [48]. The MIM-seq-treated hGFs showed more collagen than the vehicle-treated hGFs, reversing the effect caused by the inflammatory stimulus and recovering the collagen levels of the control cells.

The modulation of the proteolytic responses of the collagen caused by the inflammatory stimulus by MIM-seq at long-term treatment could be confirmed by the Sirius Red staining of collagen fibers present on the GTE tissues. Collagen networks in different tissues can be studied using Sirius Red staining under polarized light microscopy, helpful in investigating ECM-related processes in various biological and pathological contexts [49,50]. They enable the evaluation of the presence, quantity, and spatial distribution of collagen fibers. In this study, after the long-time treatment of GTEs, MIM-seq tissues presented visually higher content of larger collagen fibers as bright yellow and orange, which could indicate the effect of the treatment counteracting collagenolytic metabolism, contributing to protecting the integrity of gingival tissue in an inflammatory situation. The results of collagen deposition are in line with those obtained previously in the long-term experiment using hGF cell culture.

Regarding extracellular matrix (ECM) turnover for hGF at 24 days, although in short-time experiments some of the MIM showed an increase in MMP-1, the MIM-seq results showed that the cumulative effect could be a reduction in this enzyme, and, therefore, this is in agreement with the collagen values obtained in the MIM-seq results. However, the results for the 3D GTE turnover of MMP-1 and TIMP-1 after inducing an inflammatory stimulus manifested in a more complex system had no effect on a reduction in this enzyme. Therefore, the increase in collagen content seen through Sirius Red staining in the long-term MIM-seq treatment may not be related to the modulation of extracellular matrix turnover. An increase in collagen content after MIM-seq treatment might be explained by increased collagen production rather than decreased collagen degradation, a tendency already discussed in the previous work on the action of BMP-4 (5 CH) [23].

In summary, the sequential MI treatment MIM-seq displayed cellular anti-inflammatory effects, reducing secreted PGE2 and IL-6 levels and restoring collagen content in an in vitro model of hGF periodontitis. In a more complex 3D tissue of oral mucosa, MIM-seq promoted higher collagen content, although the anti-inflammatory effect could not be confirmed. However, a more in-depth investigation into the system that may be behind the recovery of collagen synthesis by the treated fibroblasts would be of great interest. Similarly, to gain a more complete picture of the mode of action of MIM-seq, it would be interesting to analyze its effects on an in vivo preclinical model of periodontal disease.

## 4. Materials and Methods

### 4.1. Testing the Micro-Immunotherapy Medicine 

The tested MIM-seq consisted of a sequence of 10 capsules containing pillules (sucrose–lactose pillules in each capsule) for oromucosal administration, each having a unique combination of active substances. Due to confidentiality issues arising from intellectual property, the composition of the 10-capsule sequence of MIM-seq has not been disclosed. The overall active substances employed in MIM-seq as well as the composition of the 5 tested capsules are listed in Table 1.

The manufacturing process of MI medicines has been previously explained [21,31,32] and recently illustrated [31,32].

The vehicle consisted of lactose–sucrose pillules lacking active substances and was manufactured in order to provide a proper experimental control for preclinical research.

For experiments of hGF, the pillules contained in each tested capsule were freshly diluted in 50 mL of culture medium to reach the final concentration of 22 mM of sucrose-lactose [51,52,53]. In the case of the GTE 3D culture model, the final concentration of sucrose–lactose was 44 mM.

### 4.2. hGF Cell Culture

For the experiments, primary hGFs (Provitro GmbH, Berlin, Germany) from a 27-year-old Caucasian female (lot number 313 × 100,401) were used. Provitro assures that cells are obtained ethically and legally and that all donors provide written informed consent. The cells were routinely grown at 37 °C in an atmosphere of 5% CO_2_ using fibroblast medium that consisted of Dulbecco’s modified Eagle’s medium (DMEM) low glucose (Life Technologies, Carlsbad, CA, USA), supplemented with 10% (*v*/*v*) fetal calf serum (Biosera, Boussens, France), 100 μg/mL penicillin, 100 μg/mL streptomycin (Biowest, Nuaille, France), and 50 μg/mL ascorbic acid (Sigma-Aldrich, St. Louis, MO, USA). The culture medium was renewed three times per week.

### 4.3. hGF Short-Time and Long-Time Treatment Experiments

A blind study was performed for two types of experiments: (i) a short-time experiment, where the effect of MIM-1, MIM-2, MIM-3, MIM-4, and MIM-5 on hGFs was studied after 3 days of treatment, and (ii) a long-time experiment (24 days) where the effect of the complete MIM-seq (sequence of 10 capsules) was analyzed.

In both the short-time and long-time experiments, hGFs were seeded in 48-well plates at a density of 2.0 × 10^4^ cells/well. At confluence (day 0, or D0), the cells were exposed to 1 ng/mL of IL-1β (Sigma-Aldrich) to establish the inflammatory conditions. An uninflamed, nontreated cell control and a vehicle control were run in parallel in the two experiments. Both experiments were run in six sample replicates (*n* = 6) for each group. In the short-time experiment, hGF cells were treated for 3 days with vehicle, MIM-1, MIM-2, MIM-3, MIM-4, or MIM-5. Cytotoxicity, prostaglandin E2 (PGE2), MMP-1, and TIMP-1 levels were further assessed (Figure 1A). To test the sequential medicine, the cells were treated with the vehicle or MIM-seq for 24 days. The details of the experimental plan are illustrated in Figure 1B. Readouts were obtained for: collagen deposition, detection of PGE2 and IL-6, MMP-1, and TIMP-1 levels.

### 4.4. Tridimensional Model of Gingival Tissue Equivalent (GTE) and Long-Time Treatment Experiment

The construction of the gingival tissue equivalent (GTE) followed the technique previously described [23,54], using immortalized human gingival fibroblasts–hTERT (iHGF) (Applied Biological Materials Inc., Richmond, BC, Canada) and immortalized human gingival keratinocytes (iHGK) (Gie-No3B11, Applied Biological Materials Inc.). In brief, air-lifted GTEs were treated on top of the tissue with 30 μL of 1 ng/mL IL1*β* on the first day of treatment for the establishment of the inflammation, except for the nontreated control. The air-lifted tissues were treated daily with 30 μL on top of the tissues for 10 days using freshly prepared treatments, as previously described. The experiment was run in six sample replicates *(n* = 6) for each group. The details of the experimental plan are illustrated in Figure 1C. Readouts were obtained for: (i) the detection of IL-1𝛽, PGE2, IL-6, MMP-1, and TIMP-1 levels, (ii) the immunohistochemistry of specific markers, (iii) and the Sirius Red staining of collagen.

### 4.5. Cell Cytotoxicity Assay

The cytotoxicity assay was based on the presence of lactate dehydrogenase (LDH) in the culture medium as an index of cell death. After 3 days of treatment with the vehicle, MIM-1, MIM-2, MIM-3, MIM-4, or MIM-5, the LDH activity was determined following the manufacturer’s instructions (Roche Diagnostics, Mannheim, Germany). The results were presented relative to the LDH activity of the media of cells seeded on tissue culture plastic without treatment (negative control, 0% cell death) and of cells growing on tissue culture plastic treated with 1% nonionic surfactant (Triton X-100, Sigma Aldrich, St. Louis, MO, USA) (positive control, 100% cell death), using the equation: Cytotoxicity (%) = (exp.value–negative control)/(positive control–negative control) × 100. The experiment was run in six sample replicates (*n* = 6) for each group.

### 4.6. Collagen Deposition Assessment

Ascorbic acid was used to aid collagen deposition. After 24 days of cell culture, the cells were washed with phosphate-buffered saline (PBS) and dried for 1 h at room temperature, followed by incubation for 1 h at −80 °C. Then, the cells were dried overnight at 37 °C in a humidified atmosphere, followed by 24 h at 37 °C in a dry atmosphere. The collagen was stained with 0.1% Sirius Red F3BA (Sigma Aldrich) in saturated picric acid for 1 h at room temperature. Unbound dye was removed with 10 mM HCl washes, and the dye was solubilized with 0.1 M NaOH. A microplate reader was used to measure absorbance readings at 540 nm, and the data were compared with the non-IL-1β-treated control cells which were set to 100%. Six sample replicates (*n* = 6) were run for each group in the experiment.

### 4.7. Enzyme-Linked Immunosorbent Assays

The quantification of the secreted levels of IL-1α (BMS243-2 Thermo Fisher Scientific, Waltham, MA, USA), PGE2 (EHPGE2, Thermo Fisher Scientific), IL-6 (BMS213HS, Thermo Fisher Scientific), MMP-1 (RAB0361, Sigma Aldrich), and TIMP-1(RAB0466, Sigma Aldrich) was performed using commercially available ELISA kits according to the manufacturer’s instructions.

### 4.8. Histology and Immunohistochemistry of GTE Tissues

The GTE tissues were processed for histological examination or for immunohistochemistry (IHC), as was previously described [23,54]. In addition, tissue sections were stained with Sirius Red F3BA (Sigma-Aldrich) for collagen staining. The stained tissue sections were visualized with a BX60 microscope (Olympus, Tokyo, Japan). Images were taken at 100× with a Nikon D5600 camera (Nikon, Tokyo, Japan) at a 1/250 shutter speed and ISO 12,800 under bright field and 1/40 shutter speed and ISO 8000 under polarized light for Sirius Red F3BA staining. The experiment was run in four sample replicates (*n* = 4) for each group.

### 4.9. Statistical Analysis

All data are presented as mean values ± standard error of the mean (SEM). The Shapiro–Wilk test was carried out to assume parametric or nonparametric distribution for the normality tests. The differences between groups were assessed, depending on their normal distribution, using the Mann−Whitney *U* test or the two-way analysis of variance (ANOVA) test, followed by post hoc pairwise comparisons using the LSD test. Specific computer programs were used: SPSS for Windows (version 24.0, IBM, Chicago, IL, USA) and GraphPad Prism 9 for macOS (version 9.4.1, GraphPad Software, La Jolla, CA, USA, www.graphpad.com, accessed on 5 June 2023). The results were considered statistically significant at *p*-values < 0.05. All the results were analyzed before the experiment was unblinded.

## 5. Conclusions

The results obtained in this study show, for the first time, that the sequential MI treatment, referred to as MIM-seq, effectively reduces inflammatory marker PGE2 on inflamed hGFs. MIM-seq also showed regenerative properties as well as interesting effects on collagen metabolism in the inflamed hGFs and 3D oral mucosa in vitro models of periodontitis. Therefore, in our preclinical research study, we report preliminary encouraging pieces of evidence about the in vitro efficacy of MIM-seq, which could be a promising treatment for periodontitis.

## Figures and Tables

**Table 1:** Composition of MIM-seq and of the 5 tested capsules.

MIM-seq	MIM-1	MIM-2	MIM-3	MIM-4	MIM-5
IL-1β (17 CH)		IL-1β (17 CH)		IL-1β (17 CH)	
IL-6 (17 CH)	IL-6 (17 CH)		IL-6 (17 CH)		IL-6 (17 CH)
IL-11 (15 CH)	IL-11 (15 CH)		IL-11 (15 CH)		IL-11 (15 CH)
BMP-2 (5 CH)	BMP-2 (5 CH)	BMP-2 (5 CH)	BMP-2 (5 CH)	BMP-2 (5 CH)	BMP-2 (5 CH)
BMP-4 (5 CH)	BMP-4 (5 CH)	BMP-4 (5 CH)	BMP-4 (5 CH)	BMP-4 (5 CH)	BMP-4 (5 CH)
DNA (5 CH)		ADN (5 CH)		ADN (5 CH)	
IGF-1 (9 CH)				IGF-1 (9 CH)	
GMCSF (15 CH)		GMCSF (15 CH)		GMCSF 1(5 CH)	
Na_2_SiF_6_ (3CH)		Na_2_SiF_6_ (3 CH)			
RNA (5 CH)	RNA (5 CH)		RNA (5 CH)		RNA (5 CH)
TGF-β (5 CH)					TGF-β (5 CH)
TNF-a (17 CH)		TNF-a (17 CH)		TNF-a (17 CH)	
SNA-OSTEOa-02 (18 CH)	SNA-OSTEOa-02 (18 CH)				
SNA-OSTEOb-02 (18 CH)			SNA-OSTEOb-02 (18 CH)		

Each active substance is given in CH. CH: centesimal Hahnemannian dilutions; MIM-Seq: MIM Entire Sequence; MIM-1: MIM-Tested Capsule-1; MIM-2: MIM-Tested Capsule-2; MIM-3: MIM-Tested Capsule-3; MIM-4: MIM-Tested Capsule-4; MIM-5: MIM-Tested Capsule-5; IL: interleukin; BMP: Bone Morphogenetic Protein; DNA: deoxyribonucleic acid; IGF: insulin growth factor; GMCSF: Granulocyte-Macrophage Colony-Stimulating Factor; Na_2_SiF_6_: natrum silico fluoricum; RNA: ribonucleic acid; TGF-β: transforming growth factor-beta; TNF: tumor necrosis factor; SNA: specific nucleic acids.

## Data Availability

Not applicable.

## Abbreviations

2D bidimensional model; 3D: three-dimensional; BMP: Bone Morphogenetic Protein; CH: centesimal Hahnemannian; COX: cyclooxygenase; DMEM: Dulbecco’s modified Eagle’s medium; DNA: deoxyribonucleic acid; ECM: extracellular matrix; ELISA: enzyme-linked immunosorbent assay; FBS: fetal bovine serum; GMCSF: Granulocyte Macrophage Colony-Stimulating Factor; GTE: gingival tissue equivalent; H&E: hematoxylin and eosin; hGFs: human gingival fibroblasts; HLA: human leukocyte antigen; IGF: insulin growth factor; IHC: immunohistochemistry; iHGF: immortalized human gingival fibroblasts–hTERT; iHGK: immortalized human gingival keratinocytes; IL: interleukin; LSD test: least significant difference test; LD: low dose; LM: light microscopy; MI: micro-immunotherapy; MIM-seq: sequential MI medicine; MMP-1: matrix metalloproteinase-1; MMPs: Matrix metalloproteinase; Na_2_SiF_6_: natrum silico fluoricum; PDGF: platelet-derived growth factor; PGE2: prostaglandin E2; RT: room temperature; TGF-β: transforming growth factor-beta; TIMP-1: tissue inhibitor of metalloproteinases-1, TNF: tumor necrosis factor; RA: receptor antagonist; RNA: ribonucleic acid; SEM: standard error of the mean; SNAs: specific nucleic acids; ULD: ultralow dose; Veh.: vehicle.
